# Portion control for the treatment of obesity in the primary care setting

**DOI:** 10.1186/1756-0500-4-346

**Published:** 2011-09-09

**Authors:** Rebecca L Kesman, Jon O Ebbert, Katherine I Harris, Darrell R Schroeder

**Affiliations:** 1 Mayo Clinic, 200 1st Street SW, Rochester, MN 55905, USA; 2 University of Iowa Hospitals and Clinics; 200 Hawkins Drive, E 327-1 GH; Iowa City, IA 52242, USA

## Abstract

**Background:**

The increasing prevalence of obesity is a significant health threat and a major public health challenge. A critical need exists to develop and evaluate practical methods for the treatment of obesity in the clinical setting. One of the factors contributing to the obesity epidemic is food portion sizes. Limited data are available on the efficacy of visual or tactile devices designed to enhance patient understanding and control of portion sizes. A portion control plate is a commercially-available product that can provide visual cues of portion size and potentially contribute to weight loss by enhancing portion size control among obese patients. This tool holds promise as a useful adjunct to dietary counseling. Our objective was to evaluate a portion control intervention including dietary counseling and a portion control plate to facilitate weight loss among obese patients in a primary care practice.

**Findings:**

We randomized 65 obese patients [body mass index (BMI) ≥ 30 and < 40] to an intervention including counseling by a dietitian incorporating a portion control plate or to usual care. Following initial consultation, patients in the intervention arm were contacted at 1, 3, and 5 months by the dietician for brief follow-up counseling. Usual care subjects received instructional handouts on diet and exercise. Forty-two (65%) subjects returned to have weight assessed at 6 months. Subjects in the portion control intervention had a greater percentage change (± SD) in weight from baseline at 3 months (-2.4% ± 3.7% *vs*. -0.5% ± 2.2%; p = 0.041) and a non significant trend in weight change from baseline at 6 months (-2.1% ± 3.8% vs. -0.7% ± 3.7%; p = 0.232) compared with usual care. Nearly one-half of patients assigned to the portion control intervention who completed the study reported the overall intervention was helpful and the majority would recommend it to others.

**Conclusions:**

Our findings suggest that a portion control intervention incorporating dietary counseling and a portion control plate may be effective for enhancing weight loss among obese subjects. A portion control intervention deserves further evaluation as a weight control strategy in the primary care setting.

**Trial registration:**

Current controlled trials NCT01451554

## Background

Since 1960, the prevalence of obesity has doubled in the United States and one-third of the adult population is obese (body mass index [BMI] ≥ 30.0) [1]. Obesity is associated with significant morbidity, mortality, and healthcare costs and is a leading preventable cause of death [2]. Among individuals younger than 70 years of age, overweight-obesity is estimated to be the second leading cause of preventable death [3]. Pharmacologic interventions for the treatment of obesity are limited and are associated with significant side effects [4]. Exercise and psychological interventions have been demonstrated to be effective for weight loss, especially when combined with dietary strategies [5,6].

Since portion sizes have a significant influence on energy intake [7], a portion control intervention may be a useful strategy to enhance and promote weight loss among obese patients. Food portion control interventions employ clinical strategies for increasing the awareness of portion sizes or caloric content of food using visual cues or tools as a point of reference, such as a plate demonstrating appropriate portion sizes. A randomized controlled study in Canada observed that a portion control plate led to significant weight loss and decreases in hypoglycemic medications among obese patients with diabetes [8]. Another study among obese young people discovered that a portable computerized device weighing the meal plate and providing feedback to slow the rate of food intake and reduce total intake was effective for decreasing body weight [9].

To date, no studies have been published assessing the effectiveness of portion control interventions in a primary care setting. We conducted a pilot study to assess the effectiveness of an intervention including a portion control plate and dietary counseling for weight loss among obese patients in a general medicine primary care practice.

## Methods

### Subjects

The Mayo Foundation Institutional Review Board (IRB) reviewed and approved the study protocol prior to recruitment and enrollment. Potential subjects were recruited from a primary care internal medicine practice at the Mayo Clinic in Rochester, MN. Male and female patients were eligible for enrollment if they were men and women between the ages of 18 and 75 with a body mass index (BMI) of ≥ 30 and < 40 (obesity classes II and III). Exclusion criteria included presence of active cancer, participation in an organized weight loss program, current weight loss medication, history of bulimia or anorexia, current treatment for psychiatric illness other than anxiety or depression, surgery within the 3 months before enrollment or planned during the study period, current or planned pregnancy, and a history of gastric bypass or planned gastric bypass.

### Procedures

Prior to the patient's appointment with their primary care provider, the patient's medical record was reviewed to assess BMI. Medical records were reviewed only if the patient had provided a universal research authorization to the Mayo Clinic allowing for chart reviews of this nature. If potentially eligible based on BMI, a letter was sent to the patient informing them of the study and asking them to discuss enrollment with their primary care provider at their upcoming appointment.

At the appointment, the patient was informed of the study and asked if they would like to participate. If the patient was interested, a study coordinator contacted the patient to discuss details of the study and confirm eligibility. Patients completed informed consent and were randomized. Randomization was stratified by gender using sealed envelopes. Patients were randomized to either the portion control intervention or usual care by the study coordinator.

If the patient was randomized to usual care, Mayo Clinic pamphlets entitled "Lifestyle Changes for Healthy Weight" and "Exercise: Getting Started and Staying With It" were given to the patient. These patients received pamphlets in an attempt to standardize potentially heterogeneous dietary and exercise advice received from different primary care providers. If the patient was randomized to the portion control intervention, a 60-minute appointment with a dietician was scheduled. At the dietitian appointment, a baseline assessment of weight history, nutritional intake, and exercise and non exercise activity was conducted. The dietician reviewed food choices, portion control, consistency and timing of meals, meal plans and appropriate use of snacks. The patient received specific written instructions on how to use a commercially-available calibrated portion control plate and bowl. The portion control plate was made of clear glass with black print dividing it into three sections (one one-half and two one-quarter sections). One-half of the plate was labeled "vegetables," one-quarter was labeled "fish, lean meat, chicken & nuts," and one-quarter was labeled "potatoes, pasta, rice, beans and whole grains." The bowl was clear glass with black print designating "1/3 cup," "1/2 cup," and "1 cup." The patient was instructed to use the plate for their largest meal of the day and encouraged to use the plate/bowl for all meals.

The patients were followed for 6 months following randomization. All patients were scheduled for weight assessments at 3 and 6 months. Weight assessments were conducted using digital clinic scales with shoes off and clothes worn. Patients in the portion control intervention group were contacted by phone or email (based on patient preference) at 1, 3, and 5 months by the dietitian who briefly provided additional counseling and assistance. At study completion, patients in the portion control group were provided with a survey to assess their satisfaction with the intervention.

### Statistical Methods

The primary endpoint was the percentage weight change from baseline at 3 and 6 months. Analyses were performed using an intention-to-treat approach whereby subjects were analyzed according to randomized treatment. No covariate adjustment was included in the primary analysis. We used descriptive statistics to summarize demographic data and other baseline characteristics, along with results of the satisfaction survey.

Weight change from baseline at 3 and 6 months was expressed as a percentage of baseline weight. For this study, we anticipated that the difference in weight change between groups would likely be larger at 6 months than at 3 months (i.e. time-by-treatment interaction effect) and that the variance of the weight change would likely be larger at 6 months than at 3 months. For these reasons, we decided *a priori *that for this pilot study we would perform separate analyses for the 3 and 6 month time points. Weight change was compared between groups using the two-sample t-test. We conducted analyses excluding subjects with missing data (complete case analysis) and also using the last observation carried forward (LOCF) to impute values for subjects who discontinued study participation. In all cases, the difference in weight change between groups (plate minus control) was summarized using a point-estimate and corresponding 95% confidence interval. Two-tailed p-values ≤ 0.05 were considered statistically significant.

The assumptions used for sample-size calculations were based upon previous literature [8]. Assuming a mean percentage of weight loss of 2% in the intervention group and 0% in the controls with a standard deviation of 3%, we determined that a total sample-size of 80 patients would provide statistical power (two-tailed, alpha = 0.05) of 83% to detect a difference between groups. Due to a shortage of portion control bowls which could not be reordered from the original source, the decision was made to discontinue enrollment after randomizing 65 subjects. Under the assumption that the standard deviation is 3%, an effective sample-size of 65 subjects provided statistical power (two-tailed, alpha = 0.05) of 75% to detect a difference in weight change of 2% between groups.

## Results

Sixty five patients (25 men and 40 women) were randomized. Groups were similar at baseline (Table 1). The overall mean ± SD age was 55.9 ± 10.0 years with a mean weight of 98.2 ± 12.6 kgs (range 74.7 to 129.3 kgs). Twenty-three percent of subjects (N = 15) had diabetes and 5% (N = 3) were current smokers.

**Table 1:** Baseline subject characteristics

	Usual Care N = 32	Portion Control N = 33
Age, years		
Mean ± SD	56.3 ± 10.7	55.4 ± 9.4
Range	32 to 75	31 to 74
Gender, N (%)		
Male	12 (38)	13 (39)
Female	20 (62)	20 (61)
Weight, kg		
Mean ± SD	98.8 ± 12.5	97.6 ± 12.8
Range	75.2 to 127.2	74.7 to 129.3
Education, N (%)		
High school graduate	7 (22)	8 (24)
Some college	12 (38)	14 (42)
4-year college degree or more	13 (41)	11 (33)
Work status, N (%)		
Full time	19 (59)	22 (67)
Part time	2 (6)	2 (6)
Unemployed	1 (3)	1 (3)
Retired	10 (31)	8 (24)
Current tobacco use, N (%)		
No	30 (94)	32 (97)
Yes	2 (6)	1 (3)
Diabetes, N (%)		
No	25 (78)	25 (76)
Yes	7 (22)	8 (24)
Current exercise, N (%)		
None	4 (12)	4 (12)
1 to 90 minutes per week	8 (25)	13 (39)
91 to 150 minutes per week	16 (50)	9 (27)
> 150 minutes per week	4 (12)	7 (21)

Among those who completed the study, subjects in the portion control intervention had a greater percentage weight change from baseline compared to usual care (-2.4% ± 3.7% vs. -0.5% ± 2.2%; p = 0.041) at 3 months (Table 2). A trend toward increased weight loss with the portion control intervention was observed using the LOCF analysis (-1.7 ± 3.3 vs. -0.4 ± 1.9; p = 0.062) at 3 months. Non significant trends toward increased weight loss with the portion control intervention were observed at six months (Table 2). Among participants in the portion control intervention, the percentage weight change from baseline to 6 months did not differ significantly between those who reported using the portion control plate ≥ 2 times per day compared to those using the plate < 2 times per day (-2.7 ± 3.8 vs -1.1 ± 3.8; p = 0.40).

**Table 2:** Weight change from baseline at 3 and 6 months

	Usual Care (N = 32)		Portion Control (N = 33)		Difference in Means (Plate-Control)		
				
		mean ± SD		mean ± SD			
	N	(min, max)	N	(min, max)	Estimate	(95% C.I.)	P-value*
3 months							
Change from baseline (kgs)							
Complete case	24	-0.6 ± 2.2 (-6.4, +2.3)	23	-2.2 ± 3.6 (-11.6, +4.2)	-1.6	(-3.3, +0.1)	0.071
Last value carried forward	32	-0.4 ± 1.9 (-6.4, +2.3)	33	-1.5 ± 3.1 (-11.6, +4.2)	-1.1	(-2.4, +0.2)	0.099
% change from baseline							
Complete case	24	-0.5 ± 2.2 (-5.2, +2.3)	23	-2.4 ± 3.7 (-11.4, +3.9)	-1.8	(-3.6, +0.7)	0.041
Last observation carried forward	32	-0.4 ± 1.9 (-5.2, +2.3)	33	-1.7 ± 3.3 (-11.4, +3.9)	-1.3	(-2.6, +0.1)	0.062
6 months							
Change from baseline (kgs)							
Complete case	23	-0.9 ± 4.2 (-15.7, +3.9)	19	-1.9 ± 3.7 (-10.0, +6.1)	-1.0	(-3.5, +1.5)	0.414
Last value carried forward	32	-0.5 ± 3.6 (-15.7, +3.9)	33	-1.0 ± 3.0 (-10.0, +6.1)	-0.5	(-2.2, +1.1)	0.528
% change from baseline							
Complete case	23	-0.7 ± 3.7 (-12.3, +3.7)	19	-2.1 ± 3.8 (-9.8, +5.6)	-1.4	(-3.7, +0.9)	0.232
Last observation carried forward	32	-0.4 ± 3.2 (-12.3, +3.7)	33	-1.2 ± 3.1 (-9.8, +5.6)	-0.8	(-2.3, +0.8)	0.311

* Two-sample t-test

Among the 19 patients in the intervention group who completed the study, 47% perceived that the overall portion control intervention was helpful. Sixty-eight percent endorsed that the counseling at the dietitian visit was helpful and 79% would recommend the portion plate to family or friends. Thirty-two percent reported that they used the plate for one meal per day, while 37% and 26% said that they used the plate for two or three meals per day. Forty-two percent of patients said that they would continue to use the portion plate after the study.

## Discussion

In this pilot study, we observed weight loss at three months among obese patients in a primary care practice who received a portion control intervention. Our findings are comparable to the amount of weight loss achieved in a study performed in Canada which observed a weight loss of 1.8% ± 3.9% among obese diabetics using a portion control plate compared with 0.1% ± 3.0% among the control group. In the current study, patients in the usual care group receiving two Mayo Clinic designed educational handouts had essentially no weight loss. Almost half of the patients in our study group perceived the overall portion control intervention to be helpful and the majority would suggest the portion control plate to family and friends.

The attrition rate in the current study (35%) at 6 months is higher than the mean attrition rate of 22% within 6 months in studies of behavioral interventions for obesity [10]. However, our attrition rate is significantly less than commercial weight loss programs with reported attrition rates as high as 70% at 12 weeks [11]. Our higher attrition may relate to the reduced patient interaction between three and six months. Indeed, our attrition rate at 3 months up to which time the dietician was more engaged with the portion control intervention group was 28% increasing to 35% by the end of the trial. Higher program adherence might be expected with a clinical trial involving more frequent contacts with interventionists.

Despite the fact that we had more patients in the portion control intervention drop-out, counseling by the dietitian was ranked favorably. The majority of subjects felt that the portion plate would be helpful for other people trying to lose weight and would recommend it to family members or friends interested in losing weight. However, if we assume that the drop-outs did not have a favorable response to the portion control plate then we should be less optimistic about how the plate will be perceived by obese patients in a general medical practice. Ideally, the portion control plate could be part of a menu of options that patients interested in losing weight could use. Favorable perceptions of a portion control plate could be enhanced by leveraging existing technologies and providing feedback about the speed of food consumption to enhance satiety with smaller portions [9].

Forty-two percent of patients reported that they would continue to use the portion control plate after the end of the study which seems high considering that most obese patients do not adhere to dietary programs beyond six months [12]. We hypothesize that the provision of a re-usable visual tool may increase long-term adherence to a clinical weight loss intervention. We did not conduct follow-up surveys to determine what percentage of subjects actually continued to use the portion control plate.

In June 2011, the United States Department of Agriculture replaced the food pyramid with the "food plate" [13]. The new image is a plate-shaped diagram or pie chart. This model could be leveraged to promote the use of a portion control plate in clinical practice. Interventions effectively linking the new "food plate" model to the portion control plate to promote healthy eating and weight loss are needed.

Limitations of our study include the small sample size, an inability to blind the intervention, and incomplete follow-up. In addition, we excluded morbidly obese patients and patients under current treatment for psychiatric illness other than anxiety or depression which limits the ability to generalize to the usual primary care practice. Our intervention included counseling by a dietitian, the portion control plate and bowl, and calls or emails by a dietitian. Our multifaceted intervention makes it difficult to determine the clinical effect of each individual component on weight loss. Future studies should include an additional study arm to evaluate the effect of the portion control plate with dietary counseling compared to dietary counseling alone.

Unfortunately, our study was too small to assess for gender, age effects or baseline BMI effects. Previous research has demonstrated that weight loss is more likely to occur among patients who are older, weigh less at baseline [14] and are male [12]. Future studies of a portion control intervention should evaluate these potential predictors of treatment response.

## Conclusions

Effective and practical tools to combat the epidemic of obesity desperately need to be evaluated and disseminated. Our pilot study demonstrated weight loss among obese patients receiving a portion control intervention including dietary counseling and a portion control plate in a primary care general medicine practice. Larger studies are needed to assess the utility of portion control tools in primary care and corroborate the findings of our small clinical pilot.

## List of Abbreviations

BMI: body mass index; SD: standard deviation; CI: confidence interval.

## Competing interests

The authors declare that they have no competing interests.

## Authors' contributions

RLK conceived of the study, wrote the protocol, obtained funding, coordinated subject enrollment, and drafted the manuscript. JOE assisted with study design and oversight and provided critical input on protocol design, data interpretation and manuscript writing. KIH assisted RLK and JOE with study design, study enrollment, data analysis and manuscript writing. DRS performed the randomization and conducted the data analyses. All authors have read and approved the final version.
